# Estimates of fluid intake, urine output and hydration-levels in women from Somaliland: a cross-sectional study

**DOI:** 10.1017/jns.2021.54

**Published:** 2021-08-20

**Authors:** Espen Heen, Amal A. Yassin, Ahmed A. Madar, Maria Romøren

**Affiliations:** 1 Department of Community Medicine and Global Health, University of Oslo. P.O. box 1130, Blindern, 0318Oslo, Norway; 2 Department of Women's Health, Manhal hospital, Hargeisa, Somaliland

**Keywords:** 24-h urine volume, Beverages, Dehydration, Somaliland, Total fluid intake, Urine colour, Women, BFQ, beverage frequency questionnaire, MCH, mother–child health clinics, PWI, plain water intake, TFI, total fluid intake, TWI, total water intake, Ucol, urine colour, Uosm, urine osmolality, Usg, urine specific gravity, Uvol, urine volume

## Abstract

The study objective was to measure fluid intake and associations with background characteristics and hydration biomarkers in healthy, free-living, non-pregnant women aged 15–69 years from Hargeisa city. We also wanted to estimate the proportion of euhydrated participants and corresponding biomarker cut-off values. Data from 136 women, collected through diaries and questionnaires, 24h urine samples and anthropometric measurements, were obtained with a cross-sectional, purposeful sampling from fifty-two school and health clusters, representing approximately 2250 women. The mean (95 % CI) 24 h total fluid intake (TFI) for all women was 2⋅04 (1⋅88, 2⋅20) litres. In multivariate regression with weight, age, parity and a chronic health problem, only weight remained a predictor (*P* 0.034, *B* 0.0156 (l/kg)). Pure water, Somali tea and juice from powder and syrup represented 49⋅3, 24⋅6 and 11⋅7 % of TFI throughout the year, respectively. Mean (95 % CI) 24 h urine volume (Uvol) was 1⋅28 (1⋅17, 1⋅39) litres. TFI correlated strongly with 24 h urine units (*r* 0.67) and Uvol (*r* 0.59). Approximately 40 % of the women showed inadequate hydration, using a threshold of urine specific gravity (Usg) of 1⋅013 and urine colour (Ucol) of 4. Five percent had Usg > 1⋅020 and concomitant Ucol > 6, indicating dehydration. TFI lower cut-offs for euhydrated, non-breast-feeding women were 1⋅77 litres and for breast-feeding, 2⋅13 litres. Euhydration cut-off for Uvol was 0⋅95 litre, equalling 9⋅2 urine units. With the knowledge of adverse health effects of habitual hypohydration, Somaliland women should be encouraged to a higher fluid intake.

## Introduction

Adequate hydration is paramount for health and well-being, and bodily fluid balance is a fine-tuning between water loss and total water intake (TWI). Water losses through sweat, faeces, perspiration and urine are determined by environment temperature and humidity, as well as the size, activity level and nutrient intake of the individual. Proteins and inorganic salts in the diet affect the total renal solute load and subsequently the amount of water needed to excrete these osmotic active substances and maintain homeostasis^(1,2)^.

TWI is the sum of water content in beverages, including plain water, food moisture and metabolic water. Since the latter two are difficult to measure, the sum of beverages as total fluid intake (TFI) might be a good proxy in free-living individuals. TFI equals 62–80 % of TWI, depending on culture and cuisine, and the two variables are highly correlated (*r* 0.96) in several studies^(3–8)^. Established guidelines from the USA and Europe have described adequate TWI of 2⋅7 and 2⋅0 litres, respectively, and corresponding TFI of 2⋅2 and 1⋅6 litres, as necessary for ensuring adequate hydration in adult females living in temperate climate with sedentary activity level. Lactating women should increase their fluid intake by 0⋅6–1⋅1 litres, proportional to breast milk production^(9–11)^. The validity of universal guidelines, even at the population level, is questioned with so many factors impacting fluid balance^(1)^. There are currently no international guidelines for hot climate countries.

Adequate hydration is described by a urine osmolality (Uosm) between the minimal and maximal concentrating capacity of the kidney, at the population level, approximately 50–800 mOsmol/kg^(1,2,12)^. Rising plasma osmolality beyond normo-physiological fluctuations in the range of 285–295 mOsmol/kg defines dehydration, a similar fall implies overhydration^(12)^. Consequently, using a urine concentration of 800 mOsmol/kg as a cut-off for dehydration is common, with a corresponding urine specific gravity (Usg) > 1⋅020 and a urine colour (Ucol) ≥ 7^(1,13–16)^. Adequate hydration can be further subdivided: In optimal euhydration, no active water-conserving happens. Recently, suggested cut-offs are Usg < 1⋅013 and Ucol < 4 to detect Uosm < 500 mOsmol/kg with high specificity and sensitivity^(12,17)^. Hypohydration, on the other hand, is defined as a ‘steady-state condition of reduced total body water’ with a regular increase in the kidney reabsorption of water^(5)^. Hypohydration and habitual low TFI are a common finding in the published literature and correlates in several studies^(18)^. Habitual low TFI and, especially, low plain water intake (PWI) are associated with increased risk of urinary tract infection, chronic kidney disease, recurrent kidney stones and poor glycaemic regulation, including increasing HbA1C and type 2 diabetes. Although no causation is established, the cardio-metabolic effects could theoretically be mediated through higher average circulating concentrations of vasopressin and cortisol^(5,12,14,18,19)^. An additional decline in physical and cognitive performance and impaired gastrointestinal, thermoregulatory, hemodynamic and renal functions are found in chronic dehydration^(20,21)^.

Little is known about female fluid intake and hydration in low- and lower-middle-income countries (LLMIC), particularly in hot and dry environments and in cultures distinct from Western lifestyles, such as Somaliland^(7)^. This is a paradox since heat, drought, high fertility, gender inequity and unhealthy socio-cultural traditions, combined with restricted water availability and quality, increase the risk of poor hydration. In Somaliland, voiding problems due to female genital cutting and limited access to decent public toilets may impact how much women drink during the day^(22)^. High mineral content in local groundwater poses an additional risk of increased renal solute load and less free water reserve for hydration regulation. Kidney stones are reported to be more prevalent in certain regions of the country^(23,24)^. To propose measures for optimising fluid intake of women in Somaliland, the aims of the present study were as follows:

1. To describe the use and feasibility of novel fluid intake and voiding data collection tools tailored for LLMIC.
2. To measure the fluid intake and urine volume (Uvol) of healthy, non-pregnant women from Hargeisa city in Somaliland, aged 15–69 years and associations with background characteristics and hydration biomarkers clinically available in this context.
3. To estimate the proportions and corresponding biomarker cut-off values of dehydrated, hypohydrated and euhydrated participants.

## Method

### Setting, study design and recruitment

We recruited non-pregnant women from the capital city Hargeisa, which harbours approximately 25 % of the two million female inhabitants in the country. The sampling frame was NAGAAD (https://nagaad.org/), a network of women empowering organisations (local non-governmental organisation, (LNGOs)), offering various activities such as literacy training in internally displaced people's camps to women rights groups for university students. From fifty different women groups, representing approximately 2200 women, five almost equally sized groups were purposefully selected, with the goal of capturing the range of age, socio-economic status (SES), education and living location within the city as described by the randomised female urban Somaliland Multi Indicator Cluster Survey sample^(25)^. In addition, breast-feeding women were recruited consecutively from two of the fourteen mother–child health clinics (MCH) in the city, since they were underrepresented in NAGAAD.

### Inclusion of participants

The inclusion of each woman was contingent on habitual fluid/food intake; normal fluid and energy metabolism/balance; urine collection ability and record/recall competency (detailed exclusion criteria in Supplementary Table S1). Receipts, medicine containers or medicines confirmed drug use.

### Data collection

A structured oral questionnaire in English was developed with items from pre-existing, well-tested questionnaires^(26–29)^. Two native-speaking, bilingual English/Somali health workers provided parallel translations into Somali. Background data on health and reproductive history, SES, household and eating habits were obtained in oral Somali.

Weight (in between meals) was measured at 0⋅1 kg precision (Seca 874 and 813) and height to the nearest 0⋅1 cm (Seca 217) with the women standing barefoot, covered in a standard underdress (*dirac*) and a light head cover (*shalmad*), details as described elsewhere^(30)^.

Fluid intake and voiding frequency/volumes during a 24 h trial were obtained through four interconnected methods (1-4). A detailed explanation of the methods and tools in use is found in the Supplementary material. Since general diet recall questionnaires have a tendency to underestimate TFI, a fluid specific diary and a beverage frequency questionnaire (BFQ) were developed and piloted with illiterate women^(31)^:

1. Illiterate women used a 24 h box-diary – a small box with coloured paper symbols that helped the women log number and volumes of beverages and voids during the trial. Literate women used a similar symbol-diary with six-time intervals covering 24 h.
2. In a 24 h recall interview, the diaries were investigated and the data systematised. The sum of beverage frequency and volumes became TFI, and the sum of voiding frequency with corresponding magnitude (small = 1, medium = 2, large = 3) became ‘24 h urine units’.
3. The BFQ investigated how often women were drinking plain water (fifteen types or sources), hot drinks (four types), milk and dairy products (six types), juices (four types) and soft drinks during the last 12 months. A semi-quantitative number of servings of each fluid type were recorded with the alternatives: never or less than one time per month, one to three times per month, one time per week, two to three times per week, four to six times per week, one time per day, two to three times per day, four to six times per day, more than seven times per day. This provided data for daily TFI averaged across a year (yearTFI).
4. After voiding at time zero, the women started to collect urine through the day and night and did a final void collection at the place of interview exactly 24 h later. The women were asked about urine spillage or bottle leakage, and missed voids were registered. Fruit and vegetable intake during the 24 h were assessed.

### Field work

Nine bilingual female nurses from Hargeisa were trained in research ethics and survey methodology and evaluated in use of the exclusion criteria, the questionnaire, the fluid intake and voiding diaries, anthropometry and volume measurements. Inter-assessor agreement showed fair reliability in accordance with ‘Anthropometric measurement error and the assessment of nutritional status’^(32)^. The research assistants piloted the 24 h trial.

The field work happened in May and June 2011 in close collaboration with leaders and teachers for each LNGO/MCH group. The superiors made the women feel secure and ensured good compliance. De-briefing and discussion with the whole research team were done regularly in order to improve performance.

The women were individually trained to use the tools and their competency verified. During the 24 h trial, a nurse contacted the women once to strengthen compliance and was available for sorting out problems. The 24 h trials started out on Sundays (44 %); Mondays (8 %) and Tuesdays (48 %), not coinciding with Muslim festivals.

### Laboratory analysis

The incoming temperature of the urine was between 12 and 18°C. All measurements were done by two assessors. The urine samples were screened with a human chorionic gonadotropin test. Uvol was measured by a dietary scale (Seca Culina) at 1 g precision. Ucol was assessed visually by a chart displaying eight different dilutions, light straw-yellow being 1 and dark green-brown 8. The colour chart had been validated and found suitable as a hydration biomarker tool for women in all reproductive states^(11,33)^. With a paper-white wall of 0⋅5 m behind, light from above (four 20 watt fluorescent tubes, Comet FL 20D, white, Indonesia) was passing through 30–50 ml urine in an ordinary water bottle (Durdur 750 ml bottle, local brand) and in a clear, small plastic cup (Party TechPak 200 ml). The colour chart was placed adjacent to the longest way of light horizontally through the bottle and cup. The average of four readings was recorded. A urinalysis strip (Insight Xpert, 10SW Reagent strip, Acon laboratories) provided grading for glucose, protein, pH and Usg by visual inspection in accordance with the ‘directions for use’, details in Supplementary Table S2. All strips were read in the same light conditions.

### Statistics

Sample size was guided by Uvol with a standard deviation (sd) of approximately 400 ml^(34)^. A design effect of 1⋅5 for the clusters and a clinical precision of ±100 ml were set and provided a sample size of ninety-two. Including possible skewed distribution, dropouts and post-trial exclusions, we aimed for a total enrolment of 150 women.

Missing, conflicting or irregular data were investigated with the research participants. Double data entry error (Epidata Entry version 3.1) was 0⋅26 % before corrections. Data analyses were done with Epidata Analysis version 2.2 and IBM SPSS version 26.

Uvol was calculated as urine weight/Usg. Data from the full year BFQ were all recoded into frequencies per day and mean daily volumes estimated (yearTFI).

Distributions were inspected visually and assessed with skewness and kurtosis, tests of normality and outlier checks. Clustering effects for key outcome variables were generally small (intraclass correlation coefficient <10 %) and non-significant when assessed with a linear mixed 0-model, only including the intercept with the clusters as a random variable. The data have been treated as non-clustered.

For group sample sizes of >30, means were compared with the independent-samples *t*-test, adjusted for unequal variances. For more than two comparisons, one-way ANOVA with *post hoc* Tuckey's test was used. The non-parametric Mann–Whitney *U* test or, for more than two samples, the Kruskal–Wallis test was used with non-normal distributions of the differences. Categorical data were compared with Pearson's *χ*^2^ test or Fisher's exact test.

In Pearson's product-moment correlation, we used square root and log_10_ transformations to ensure best fit to normality, meeting assumptions of linear relationships and homoscedasticity. Usg and Ucol were analysed with Spearman's rank-order correlation (*ρ*). Univariate and multivariate regression honoured the assumptions of linearity, no co-linearity, and independence, normality and homoscedasticity of residuals. The significance level (*α*) was 0⋅05.

### Ethics

Research permission from the Ministry of Health in Somaliland (No. MOH/3c11.00/2011) was granted on 11 January 2011. Ethical clearance was given by the Norwegian Regional Ethical Committee (No. 2010/1905 REK Sør-Øst) on 15 September 2010 and extended until 2022.

Information was given orally and in writing with special emphasis on the voluntary character of participation and the possibility of withdrawal at any time during the study without any consequences. Oral consent was observed by a third party – often a leader from her LNGO – and signed. Discretion and confidentiality were a priority during the interviews and trial. The participants received counselling on medical issues. Possible pathology, like diabetes or kidney disease, was acted on by offering the women further investigations and a 3 month treatment free of charge. A gift of 10 dollars value was given.

The ethical aspects of the study were guided by ‘the WMA Declaration of Helsinki 2008’; ‘UNESCO, IBC on Consent, 2008’; ‘Nuffield Council on Bioethics “Research in Developing Countries 2002”’ and the ‘CIOMS International Guidelines for Biomedical Research (2002) and Epidemiological Studies (2008)’.

## Results

**Fig. 1.**
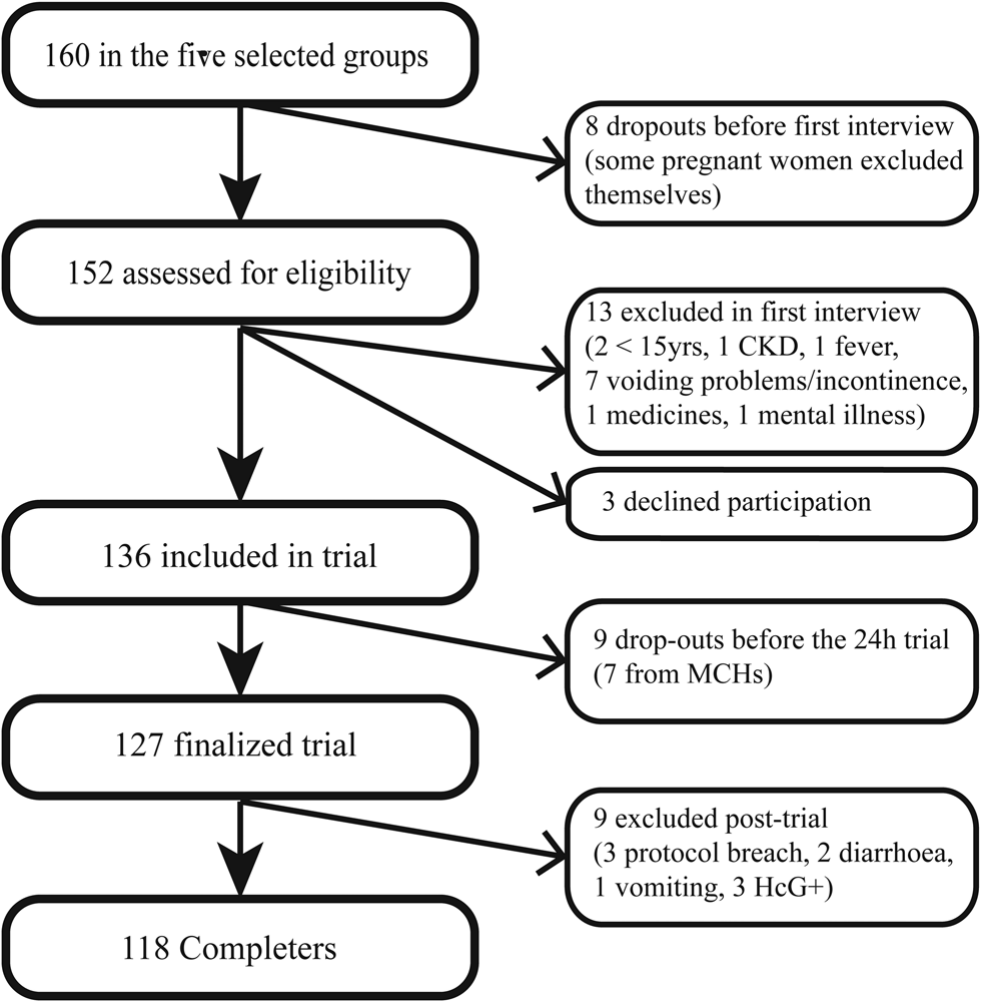
Recruitment outcome with dropouts, declined and excluded from analyses.

The pre-selected groups contained 160 women. After exclusion, 127 women (15–69 years) completed the study, a participation ratio of 79 % (Fig. 1). There were no dropouts or exclusions above 50 years of age.

Background characteristics are described in Table 1. A chronic health problem other than the exclusion criteria was reported by 46 %. There was a spread in the number of births from 0 to 18, 23 % were breast-feeding and 41 % were never married and nulliparous. The median age of breast-feeding children was 5 months (range 0–20), the majority fed four to eight times (22 %) or more than eight times (70 %) per day. In total, 97 % of the women used their memo-tool according to the instructions. Change from normal routine was reported by 10 %. Through the 13 d of trials, the mean (min.–max.) 24 h temperature was 25 (24–26°C), and the relative humidity was 58 (52–65%). Supplementary Table S3 describes background characteristics of the various subgroups with *P*-values of group differences. The declining, dropped out and excluded women were not significantly different from the completers.

**Table 1.** Background characteristics of 118 women from Hargeisa city

	**Mean**	**sd**
Age (years)	30⋅2	12⋅6
Weight (kg)	60⋅4	13⋅6
Height (cm)	161⋅1	5⋅8
Household size (*n*)	8⋅6	3⋅1
Children 0–14 years in house (*n*)	3⋅3	2⋅3
		
	**Median**	**IQR**
Number of childbirths	2⋅5	0–8
		
	**%**	***n***
<22 years	37⋅3	44
22–34 years	29⋅7	35
≥35 years	33⋅1	39
BMI <18⋅5	16⋅1	19
BMI 18⋅5–24⋅99	48⋅3	57
BMI 25⋅0–29⋅99	27⋅1	32
BMI <30⋅0	8⋅5	10
Chronic health problem	45⋅8	54
One or two meals in the trial	12⋅7	15
Three or more meals in the trial	87⋅3	103
Never attended school^a^	48⋅3	57
Divorced/widowed	8⋅5	10
Single	40⋅7	48
Married	50⋅8	60
Man head of household	69⋅5	82
Ahmed Dhagax district	20⋅3	24
M. Hayb/M Mooge distr.	29⋅7	35
Gacan Libaax distr.	0⋅0	0
26th June distr.	13⋅6	16
Ibrahim Koodbuur distr.	36⋅4	43
Hut	27⋅1	32
Iron sheet house	31⋅4	37
Cement/brick/stonehouse	41⋅5	49
Electricity in house	47⋅5	56
Total	100⋅0	118

IQR, Inter quartile range.

a Minimum schooling defined as a 6 month full-time course or a successful literacy course.

### Fluid intake

The median (interquartile range (IQR)) TFI of the women during the trial was 1⋅87 (1⋅22) litres. We found significantly different mean TFI for age, a chronic health problem and parity (dichotomous) (Table 2). SES variables, like school inclusion, completed educational level or housing standard, were not associated with TFI. In univariate regression, weight predicted TFI (*F* 18⋅24, *P* < 0⋅005, adjusted *R*^2^ 0⋅13) with a positive slope (*B* 0⋅0242, *P* < 0⋅005), indicating a 24 ml increase per kg bodyweight. BMI was also a positive predictor with an adjusted *R*^2^ of 0⋅12. In multivariate regression (adjusted *R*^2^ 0⋅14, ANOVA: 4, 5⋅62, *P* < 0⋅0005) with the significant univariate predictors, only intercept [*B* (95 % CI) 0⋅79 (0⋅41, 1⋅53) litres, *P* 0⋅04] and weight remained significant [*B* (95 % CI) 16 (12, 30) ml, *P* 0⋅034].

**Table 2.** Subgroup comparison of mean TFI and Uvol during 24 h, 118 women in Hargeisa

		TFI (litres)			Uvol (litres)		
	*n*	Mean	95% CI	*P*-value	Mean	95% CI	*P*-value
All	118	2⋅04	1⋅88–2⋅20		1⋅28	1⋅17–1⋅39	
<22 years	44	1⋅83*	1⋅60–2⋅06	0⋅022	1⋅22	1⋅05–1⋅40	0⋅577
22–34 years	35	1⋅95	1⋅66–2⋅24		1⋅26	1⋅08–1⋅44	
≥35 years	39	2⋅35*	2⋅02–2⋅68		1⋅36	1⋅13–1⋅59	
LNGO	94	1⋅96	1⋅79–2⋅14	0⋅063	1⋅21	1⋅09–1⋅33	0⋅010
MCH	24	2⋅34	1⋅92–2⋅76		1⋅56	1⋅27–1⋅85	
Chronic health problem	54	2⋅26	1⋅98–2⋅55	0⋅014	1⋅37	1⋅17–1⋅56	0⋅161
None	64	1⋅85	1⋅67–2⋅03		1⋅21	1⋅08–1⋅33	
Nulliparous	48	1⋅69	1⋅51–1⋅88	<0⋅005	1⋅12	0⋅99–1⋅25	0⋅009
Parous	70	2⋅28	2⋅05–2⋅51		1⋅39	1⋅23–1⋅55	
Breast-feeding	27	2⋅29	1⋅90–2⋅67	0⋅113	1⋅46	1⋅18–1⋅74	0⋅143
Non-breast-feeding	90	1⋅98	1⋅80–2⋅16		1⋅24	1⋅12–1⋅36	
Primary/secondary school	15	2⋅24	1⋅64–2⋅84	0⋅352	1⋅35	0⋅97–1⋅73	0⋅636
Informal^a^ schooling	103	2⋅01	1⋅83–2⋅18		1⋅27	1⋅15–1⋅39	
Nomadic hut	32	2⋅03	1⋅66–2⋅40	0⋅807			
Iron sheets house	37	1⋅97	1⋅72–2⋅22				
Cement/bricks/stone house	49	2⋅1	1⋅83–2⋅36				

a Koranic and adult literacy.

* Significant difference in a *post hoc* test.

For all types of beverages, an all-year mean (sd) of 8⋅1 (3⋅17) servings per day was found. Pure water, Somali tea and juice from powder or syrup represented 49⋅3, 24⋅6 and 11⋅7 % of yearTFI, respectively. Local drinking water made up 89⋅6 % of all servings with a mean of 1⋅83 litres/d. Mean intake of all other beverages totalled 0⋅21 litre with large individual differences and skewed distributions (Table 3). Public tap water and water from tanker trucks, providing deep or shallow groundwater, respectively, were used by 95⋅0 % of the women, while rainwater was the main source in 2⋅5 %. Practically, all had the same water sources for drinking and cooking (99⋅2 %). Drinking water was treated by 10⋅0 %. The water source supply was described as reliable or most reliable in 95⋅8 % of completers. We found a moderate positive Spearman's correlation of 0⋅46 (*P* < 0⋅005) between TFI and yearTFI.

**Table 3.** Estimated^a^ mean daily beverage intake (yearTFI) in ml derived from the BFQ, 118 women in Hargeisa

		P05	P25	P50	P75	P95	Mean	Groupmean	%
Local water	Household water, local sources	4	630	630	1260	1890	957	1830	89⋅6
	Somali tea	4	252	630	630	1260	497		
	Other types of tea	1	1	1	1	1	3		
	Coffee	4	4	4	17	36	14		
	Soup	4	4	17	36	90	35		
	Powder milk	1	1	1	36	630	78		
	Juice from powder or syrup	4	36	252	252	630	239		
	Grain porridge	0	0	0	0	2	7		
Manufactured beverages	Bottled water	4	4	4	17	252	41	92	4⋅5
	Soft drinks	2	2	2	2	36	14		
	Long-life milk	1	1	1	1	36	13		
	Imported juice	4	4	4	17	180	25		
Local dairy products	Local fresh cow's milk	4	4	4	17	252	43	77	3⋅8
	Local fresh goat's milk	2	2	2	2	18	11		
	Local fresh camel's milk	4	4	4	4	18	11		
	Local fresh yogurt	4	4	4	4	36	12		
Local juice	Fresh orange juice	2	2	2	2	90	18	42	2⋅1
	Other types of fresh juice	4	4	4	17	180	25		
Total		870	1424	1965	2501	3737	2042		100

P, Percentiles.

a Assuming that mean fluid intake and the size of drinking containers in the trial and the full year are the same, resulting in an average container volume of 252 ml.

### Urine

Looking at the temporal precision in 24 h urine collection, 93 % were within the range of −7 to +8 min. Compared with crude Uvol, adjustments for collection duration and the number of missed urine units (voids) during the trial changed mean and median upwards 1⋅51 and 1⋅41 %, respectively.

Median (IQR) Uvol was 1⋅16 (0⋅86, 1⋅59) litres. We found significantly different mean Uvol for parity, but not for age (Table 2). Two urine samples were red-tinged, but not due to beetroot, the only consumed vegetable known to affect Ucol. One woman had 1+ on protein, none had glycosuria and seven had urine with pH 7, the rest pH 5 and 6.

### Correlation between fluid intake and urine hydration biomarkers

We found strong positive linear correlations between TFI and urine volume variables and negative medium correlations of TFI with Ucol and Usg (Table 4). A sensitivity analysis, only including participants with all voids collected, did not improve the correlation coefficients significantly (not shown). The strongest linear predictor for TFI and Uvol, explaining 45 and 75 % of the variability, respectively, was 24 h urine units. Uvol regressed on 24 h urine units (*F* 372, *P* <0⋅0005) provided the equation (95 % CI): Uvol = 0⋅124 (0⋅11, 0⋅14) × urine units – 0⋅055 litre, with intercept not statistically significant from 0. Among the urine concentration variables, Ucol had the highest explanatory values with 14 % for TFI and 65 % for Uvol.

**Table 4.** Correlation coefficients matrix of fluid intake, urine volume and hydration biomarkers in 116 women

		TFI	yearTFI	24 h urine units	Uvol	Ucol	Usg
TFI	Pearson's *r*	1	0⋅489**	0⋅670**	0⋅593**	−0⋅368**	−0⋅270*
yearTFI	Pearson's *r*	0⋅489**	1	0⋅385**	0⋅272**	−0⋅093	−0⋅034
24 h urine units	Pearson's *r*	0⋅670**	0⋅385**	1	0⋅868**	−0⋅646**	−0⋅487**
Uvol	Pearson's *r*	0⋅593**	0⋅272**	0⋅868**	1	−0⋅809**	−0⋅694**
Ucol	Spearman's *ρ*	−0⋅368**	−0⋅093	−0⋅646**	−0⋅809**	1	0⋅746**
Usg	Spearman's *ρ*	−0⋅270*	−0⋅034	−0⋅487**	−0⋅694**	0⋅746**	1
	*n*	116	116	116	116	115	88

Correlations >0.50: darker colour indicates stronger correlation.

** Correlation is significant at the 0⋅001 level (two-tailed).

* Correlation is significant at the 0⋅01 level (two-tailed).

### Relative index of hydration

The strong correlation between the urine hydration variables provided support for a multi-biomarker hydration analysis. Placing the women in seven percentile groups based on increasing Uvol, we calculated the mean values for all groups in order to compare the different hydration variables. Even with group numbers as low as *n* 10, the high correlation and relatively low sd within each group ensured increasing mean values from group 1 to group 7, with one exception within TFI (Supplementary Table S4 of Supplementary material). Mean values were converted to lower cut-offs by finding the midpoint between adjacent means. Table 5 displays these values for different levels of hydration in university women from the USA (Ref.), compared with our Somaliland sample^(4)^. Using a threshold of approximately 500 mOsmol/kg, equal to Usg < 1⋅013 and Ucol < 4 as euhydration limits, the four highest Somaliland percentile groups (4–7) are euhydrated, approximating 60 % of the women. A sensitivity analysis excluding urine with pH 7 did not alter the proportions. Dehydrated women were found in the 9 % with the most concentrated urine: 5 % had Usg > 1⋅020 and concomitant Ucol > 6.

**Table 5.** Relative hydration index with lower cut-off values across seven percentile groups displaying Uvol, Usg, Ucol and TFI from Somaliland women compared with identical hydration indices (Ref.) from Armstrong *et al.*^(4)^

			Lower cut-off for hydration groups								
		Hydration categories	Uosm	Uvol (litres)		Usg		Ucol		TFI (litres)	
Group	Percentile	Ref.	Ref.	Ref.	S. land	Ref.	S. land	Ref.	S. land	Ref.	S. land
7	91–100		320	2⋅07	2⋅22	<1⋅008	1⋅007	<3	<2⋅1	2⋅80	2⋅83
6	76–90	Hyperhydration	382	1⋅83	1⋅67	1⋅008	1⋅009	3	2⋅6	2⋅47	2⋅42
5	61–75		548	1⋅24	1⋅30	1⋅012	1⋅010	4	3⋅3	1⋅83	2⋅09
4	40–60	Euhydration	705	0⋅95	1⋅05	1⋅016	1⋅012	5	3⋅9	1⋅30	1⋅88
3	25–39		809	0⋅83	0⋅84	1⋅021	1⋅014	5	4⋅6	1⋅15	1⋅68
2	10–24	Hypohydration	863	0⋅53	0⋅59	1⋅024	1⋅018	6	5⋅0	0⋅95	1⋅35
1	0–9		>863	<0⋅53	<0⋅59	1⋅026	>1⋅018	>6	>5⋅0	<0⋅95	<1⋅35

**Fig. 2.**
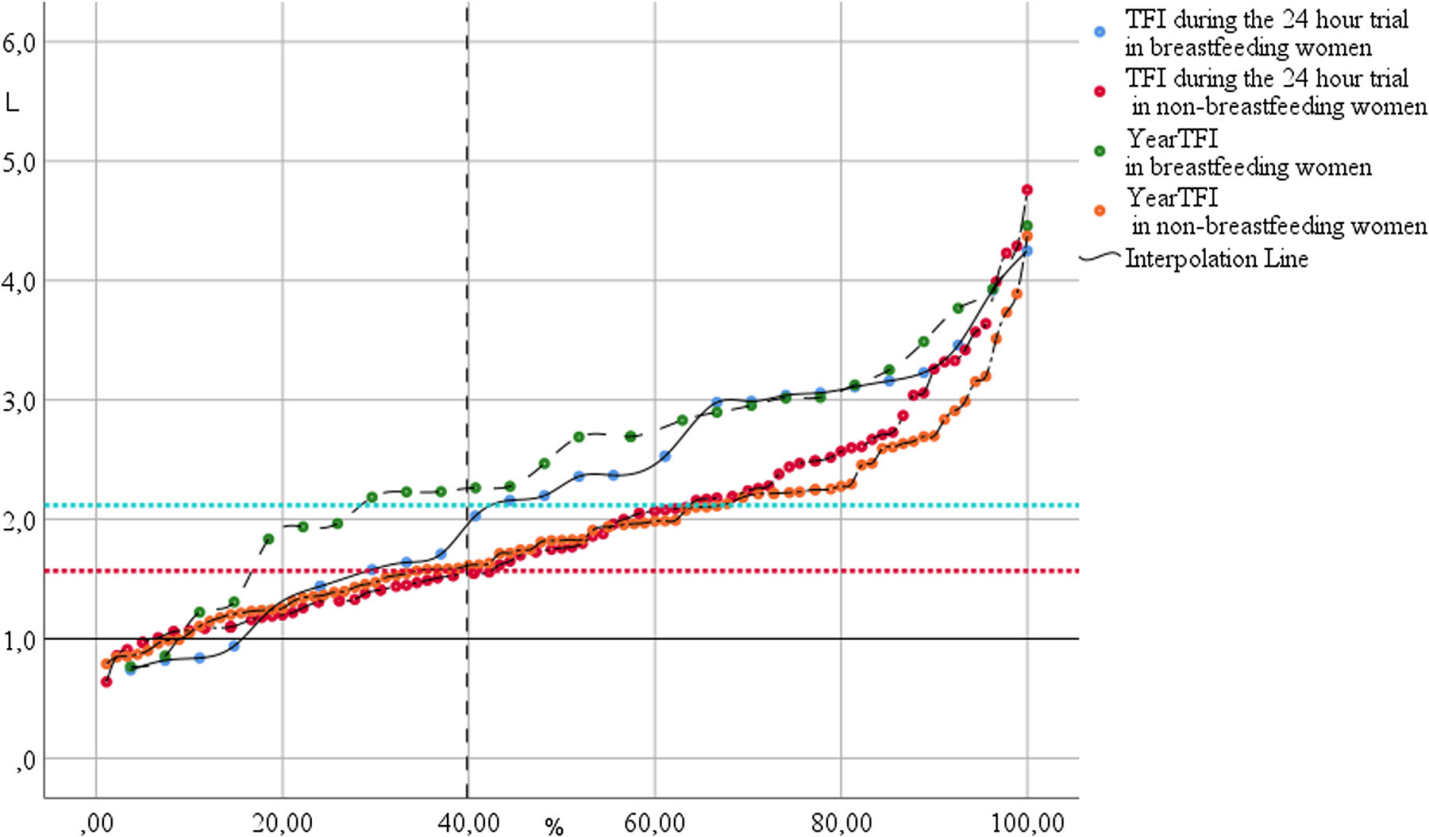
Overlay scatterplot of individual total fluid intake in litre (*y*-axis) split into breast-feeding and non-breast-feeding women. Percentiles on the *x*-axis. The two horizontal lines indicate the combined 40 percentile cut-off values of the 24 h trial (TFI) and estimates from the whole year (yearTFI) in non-pregnant, non-breast-feeding and breast-feeding women.

TFI lower cut-offs for euhydrated, non-breast-feeding and breast-feeding women in this study, using the 40–60 percentile group, are 1⋅77 and 2⋅13 litres, respectively. Similarly, plain TFI 40 percentile cut-offs are 1⋅55 and 2⋅07 litres, and yearTFI 1⋅62 and 2⋅27 litres, respectively (Fig. 2). Euhydration cut-off for Uvol is 0⋅95 litre, equalling 9⋅2 urine units.

## Discussion

This is the first study describing details of fluid intake and hydration biomarkers in healthy, free-living African women, expanding the understanding of female fluid turnover in a global context. We have shown that such a study is feasible with new data collection tools developed and piloted to ensure that illiterate women are able to provide credible information about their drinking and voiding habits. The study monitored the completeness and accuracy of data collection thoroughly. The use of urine collected through 24 h avoids coincidental fluctuation in hydration and is more reliable than spot urine measures^(35)^. The recruitment base for the women was broad, and the background characteristics of the non-completers did not differ significantly from the completers.

### Fluid intake

In non-pregnant, non-breast-feeding women from urban Somaliland, we found a mean TFI of 1⋅98 litres. A recent systematic literature review of TFI presents fifteen populations of adult females from Europe, China and the Americas with a mean TFI range of 1⋅05–2⋅89 litres, six of them above 2⋅0 litres^(7)^. A female Indonesian sample had a mean TFI of 2⋅76 litres^(36)^. In our study, TFI in lactating women was 2⋅29 litres, while French lactating women had a TFI of 2⋅10 litres^(37)^. Comparatively, the TFI of Somaliland women is quite modest. In multivariate regression, only weight remained significantly associated with TFI, as has been found elsewhere^(19)^.

Mean PWI was just below 50 % of TFI with a mean volume of 1⋅0 litre. Compared with the mean range of 0⋅5–2⋅0 litres from the twelve relevant studies in the review, Somaliland women are found in the middle, including the ratio^(7)^. However, a median PWI of 0⋅63 litre – less than three servings per day – leaves a small free water reserve to protect against dehydration on hot and active days, and we recommend that these women exchange some of their beverages for plain water.

A median of 2⋅5 cups a day of Somali tea is in line with previous findings from the country^(27)^. In the review, seven European countries have documented female tea/coffee intake of around half a litre^(7)^.

Fresh dairy products are in little use; 75 % of the women are drinking an average of 27 ml or less per day. When including powder milk, the volume is 63 ml or less, not accounting for what is usually mixed in the tea. A similar situation can be seen with fresh fruit juices: the majority have one serving only twice a month or less. In various European populations, these beverage intakes are three to ten times higher^(7)^. Milk and fresh fruits usually have a high content of micronutrients, and the women would have benefitted from a higher intake, although cost might be a challenge. We believe the female nomadic population has a higher intake of fresh milk.

### Hydration biomarker correlation

The correlation matrix describes coherence between reporting (yearTFI, 24 h urine units) and reality (TFI, Uvol) during the trial in the life of these women. The moderate correlation between yearTFI and TFI is reasonable, with expected day-to-day and seasonal variations in TFI and typical recall bias in the BFQ. Twenty-four hour urine units showed the strongest correlation with TFI (*r* 0.67) and Uvol (*r* 0.87). Our findings are interesting, since measurement only requires the sum of magnitude of voids during 24 h. The same strategy has recently been used for assessing Ucol directly in lavatories^(38)^, and the number of voids is used to assess hydration^(5,14,39)^. In this population, 24 h urine units could be used as an easy proxy for Uvol when volume scaling of urine metabolites is necessary, one urine unit (small void) equalling 124 ml of urine. No urine hydration biomarker was well suited to predict TFI.

### Hydration status

Hydration might be difficult to assess^(12)^. Uvol, Usg and Ucol are all biomarkers able to distinguish euhydration from hypohydration and mild dehydration, but accurate interpretation is context-dependent^(8,12,21)^. In this study, 24 h Uvol, Usg, Ucol and TFI represent operational biomarkers available and replicable in field work and low resource contexts^(8,21,40)^. Since Usg measures the weight of particles dissolved, and Uosm measures the number (mol), variation in the relative concentration of particles with large (protein, glucose and urea) and small (Na^+^, K^+^ and Cl^−^) molecular weights will affect cut-off levels of Usg relative to Uosm^(41–43)^. Nevertheless, studies have shown a strong correlation between the two in adults (*r* 0.94–0.99) with refractometry^(42,44)^, less so with dipstick (*r* 0.78)^(44)^. The dipstick Usg in this study measures ionic concentration. In healthy adults, protein fluctuations are only affecting measurements when the proteins are charged: pH biases the readings in a fairly linear manner, but is not systematically adjusted for in the readings of most dipsticks. Since the development usually is based on regression lines with refractometry, and individual deviation from gold standard can be high, the Usg values works better to evaluate groups than individuals^(41)^. In our study, a sensitivity analysis excluding urines with pH 7 did not alter the results, and proteinuria was not a problem.

The characteristic Ucol is mainly caused by the yellow pigment urobilin. The concentration of urobilin in urine is not only determined by hydration, but also by the type of microflora in the gut and the total amount of degraded haeme, of which Hb constitutes 80–85 %^(45,46)^. Visual Ucol assessment can be biased by individual colour and light perceptions, light conditions, size and depth of urine containers, flavonoids from food, food dyes and drugs^(16,43,47)^. Despite this, Ucol has been shown to correlate strongly with Uosm in several studies (*r* 0⋅62–0⋅88)^(4,11,16,39)^. We did not find any association between strong coloured food items and atypical Ucol in our study.

A relationship between locally available hydration biomarkers has been established in this study. We decided to use the means of hydration percentile groups as a basis for the hydration indices, reducing the impact of methodological challenges and individual variability in the biomarkers of choice. We sorted individuals into the different percentile groups based on Uvol, the only urine ratio variable. The percentile groups were wider towards the median in order to model a typical population distribution, corresponding to a similar display made by Armstrong *et al.*^(4)^. In this way, we were also able to make a relative comparison between a Western, well characterised female hydration sample and our novel study from the Horn of Africa.

Using Uvol as a contrast, Somaliland women in this study have relatively higher TFI and their urine is more diluted than the US women. A higher TFI is reasonable due to heat-related water losses, although we do not have any meteorological data for comparison. There are several explanations for the comparatively less concentrated urine apart from the methodological issues. Somaliland women might have less solutes to excrete^(1)^. A likely candidate is urea due to low protein intake, as has been shown in several African populations and a micronutrient study from Somaliland^(27,48)^. The low dairy intake does not improve protein intake in this population. Somaliland women might also have less haeme to excrete due to a consistent low Hb in the female, fertile population with a national mean of 7·4 mmol/l measured in 2009^(27)^. We are not aware of any studies that have investigated the theoretical correlation between Hb and Ucol values.

With these uncertainties in mind, we have estimated the number of dehydrated individuals to 5 % and a further 35 % as hypohydrated. Comparatively, in two female populations from Europe and US, 19 and 9 % were classified as dehydrated, respectively^(3,4)^. In US NHANES 2009–2012, more than 70 % were hypohydrated or dehydrated^(19)^. One explanation for the low dehydration level in our study might be the high availability of drinking water, which was better than we had expected. This applies to the city of Hargeisa, but should not be extrapolated to other areas of Somaliland. Another contributing factor might be the habitual high intake of Somali tea.

Using the hydration indices table, we estimated a minimum daily TFI for euhydration in this population to be 1⋅55–1⋅77 litres for non-breast-feeding women, increasing to 2⋅07–2⋅27 litres for breast-feeding women. The widespread mixed breast-feeding habits might explain the low additional TFI of 0⋅5 litre^(49)^.

There are some limitations to this study. Although the correlation matrix shows strong relationships between hydration biomarkers collected with different tools, these tools are not validated towards gold standards of Uosm and 7 d drinking diaries with direct volume measurements. The 24 h trial happened right after the rainy season, and this might have improved the availability of water and other beverages. Finally, we cannot claim that the sample is fully representative for women in Hargeisa, and the findings should not be generalised to other parts of the country or to diseased women.

## Conclusions

This article is contributing to the validation of hydration indices in a healthy, African female population with context, drinking habits, cuisine and nutrient intake different from typical populations from which current international guidelines of fluid intake and hydration have been developed. In this mixed population of non-pregnant women, TFI and PWI are comparable to a number of European populations. Intakes of fresh dairy and fruit products are almost negligible with large distributional differences. Twenty-four hour urine units is a promising variable for urine volume scaling. Assessing hydration with urine units, dipstick Usg and Ucol have good discriminatory ability at the group level and could be explored at the individual level with sensitivity and specificity against Uosm in a future study. We estimated 60 % of the women to be above the euhydration lower cut-off when using the total information in the hydration biomarker indices. Forty percent hypo- and dehydrated women are less than findings from several Western countries, and less than we had expected. The adjacent euhydration cut-off levels for TFI, Uvol and urine units can be used as a guide at the population level in similar climatic and dietary conditions. With the growing knowledge of long-term health risks, not only in dehydration, but also in habitual hypohydration, Somaliland women should be encouraged to a higher TFI and, especially, PWI. The findings are practically relevant to the health system, public health authorities and NGOs involved in nutrition and water provision in Somaliland.

## Supplementary material

For supplementary material accompanying this paper visit http://dx.doi.org/10.1017/jns.2021.54. click here to view supplementary material
