# Supplementary material for: Estimates of fluid intake, urine output and hydration-levels in women from Somaliland: a cross-sectional study
Source: J Nutr Sci. 2021 Aug 20;10:e66. doi: 10.1017/jns.2021.54 (PMC8411265; doi:10.1017/jns.2021.54)
Supplement: Supplementary file 1 [file S2048679021000549sup001.docx]

**Estimates of fluid intake, urine output and hydration-levels in women from Somaliland: A cross-sectional study**

**– Supplementary material**

**Table S1.** Detailed exclusion criteria for research subjects in the study

**1) Acute disease at the time of enrolment or during the trial**

a. Increase in frequency of defecation with non-solid stool (diarrhoea).

b. Any disturbing symptom of 1) Supra pubic pain 2) Loin pain 3) Unusual pain when voiding 4) More frequent voiding than usual 5) Urge AND one clear biochemical sign (two if biochemical signs alone) of nitrite, leukocytes or blood in urine (urinary tract infection).

c. Severe menstrual bleeding.

d. Fever (more than 38.0 °C), generalized body pain, weakness, nausea or vomiting - affecting activities in daily living.

**2) Anorexia or nausea > 1 week at the time of enrolment**

**3) Severe chronic disease, specified as**

a. Heart disease with episodes of pitting oedema (heart failure).

b. Active or end-stage liver disease.

c. Sub-acute or chronic kidney disease with one or more of the following characteristics: Haematuria, oedema, frothy urine, two or more episodes of kidney stones in the last year, glomerular filtration rate (GFR) reduced more than 50%.

d. Cancer with increased need for rest during the day compared to pre-disease state.

e. Active rheumatoid/autoimmune disease with a generalized, systemic, inflammatory reaction.

f. Endocrine disorders (Addison, Cushing or Diabetes) not treated or poorly regulated.

g. Infectious disease with a generalized, systemic, inflammatory reaction.

h. Difficulties with swallowing.

i. Voiding problems: fistula, uterine descends, incontinence, urge-symptoms, voiding duration more than 3-4 minutes.

**4)** **Physical, mental or sensory impairment not compatible with taking part in the study**

**5) Medicine use**

a. Diuretics: Thiazides, loop-diuretics and spironolactone

b. Systemic glucocorticoids

**6)** **Pregnancy**

*Methods and tools in data-collection*

Fluid intake and voiding frequency/volumes during a 24-hour trial were obtained through four interconnected methods (I-IV). A fluid specific diary and a beverage frequency questionnaire (BFQ) was developed with the support of students from the University of Hargeisa, school of Medicine, and piloted with illiterate women:

**(I)** Illiterate women used a 24-hour **“box-diary”** - a small box and two zip bags containing 20 standardized elongated blue paper-strips and 20 yellow paper-balls. For each serving, a strip was put into the box. The length corresponded roughly with how much fluid had been drunk, e.g. drinking half a cup corresponded to a half-length strip. Similarly, for each void, one, two or three balls for small (<150ml), medium (150-300ml) or large (>300ml) urine volumes, respectively, were collected and the sum constituted “24h urine units”.

The literate women used a **“symbol-diary”** (**Fig S1**) with six time intervals covering 24 hours. Four different “calls for prayer” and one cultural snack time divided the intervals. Drinking volumes were recorded by proportional colouring of up to four empty “symbol cups”. Colouring open “drops” within markings of 1/3, 2/3 and 3/3 indicated the volume/size of each void, similar to the levels for the “box-diary”. The women coloured extra drops if voiding more than twice within a time interval.

**II)** **In a 24-hour recall interview** each paper-strip, paper-ball or coloured symbol (fig S1) had to be accounted for within each time interval. Incomplete or erroneous use of the diaries was investigated and corrected. Fluid volumes were described with the proportions 1, 3/4, 2/3, 1/2, 1/3, 1/4 or 1/8 of each beverage container. The full volume of the containers used during the trial was measured to its nearest 10 ml. For bottles, the labelled volume was used. The women could also identify the actual containers from pre-measured models displaying a predominant collection of cups, glasses and bottles for water and soft drinks available in Hargeisa. If any of this failed, she was asked to give an estimate of the containers full volume in units of “dare” - a standard size cup used for measuring milk at markets, equalling approximately 220 ml. A full drinking container was defined with a fluidless rim of 0.5-0.7cm.


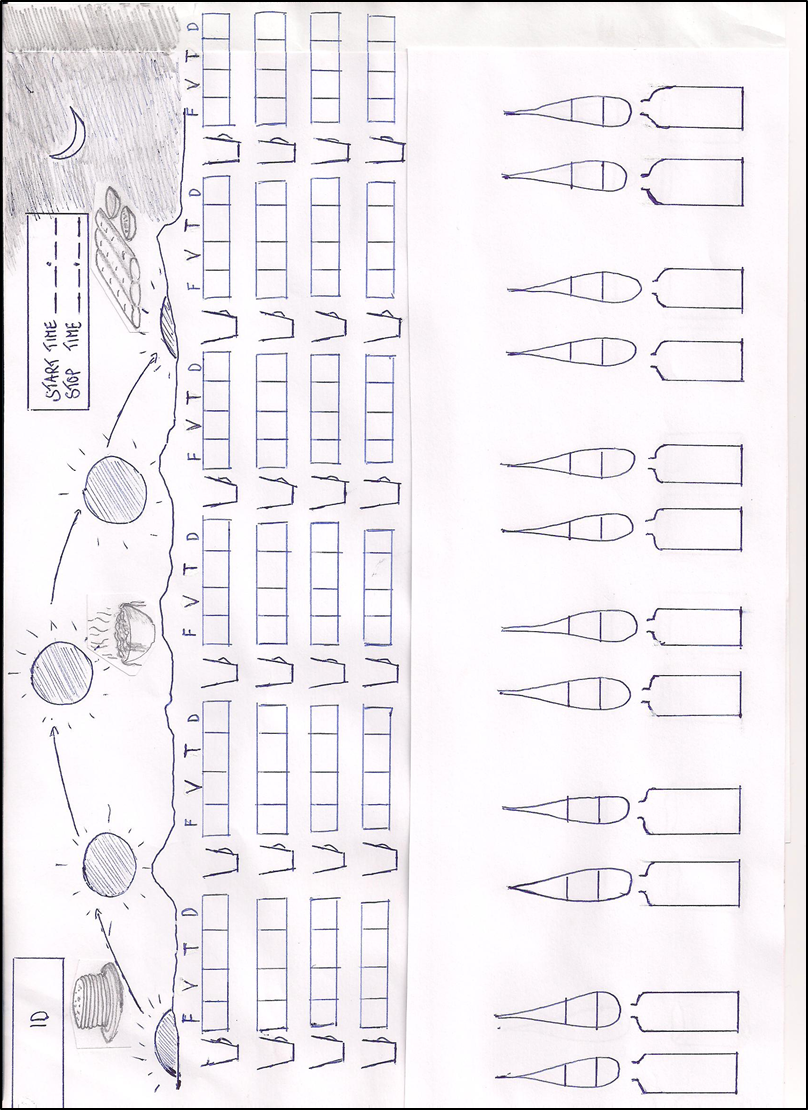


**Fig S1.** Symbol diary in the 24h trial. The women coloured the open cups (beverage) and elongated drops (urine voids). The adjacent labels were used during the final interview. F = proportion drunken, V = direct measurement of full drinking container, T = model-container volume, D = local volume unit “Dare”. The product of *proportion* and *container volume* equalled the drunken volume for each serving.

**III) The Beverage Frequency Questionnaire** (BFQ) investigated how often women were drinking plain water (15 types/sources), hot drinks (four types), milk and dairy products (6 types), juices (4 types) and soft drinks. Other alternatives could be specified. Based on the last year, a semi-quantitative number of servings of each fluid type was recorded with the alternatives: Never or less than one per month, 1-3 times per month, 1 per week, 2-3 per week, 4-6 per week, 1 per day, 2-3 per day, 4-6 per day, 7+ per day.

**IV)** **Urine collection** of 24 hours duration (Uvol) was accomplished by providing each women with a soap, tissue paper, a plastic bag, a small towel, a beaker with marks at 150 and 300ml, a funnel, a bottle of 500ml and a large urine container of 2.5 liter (Ashut Engineers Limited, Nairobi). The latter were mimicking yellow palm-oil cans in regular sale. Every woman received a large woman bag where the smaller equipment could be hidden when she collected urine outside of her home. The set-up let the woman continue with her daily activities during the 24-hour trial. The urine container was stored in a cool, dark place.

**Table S2.** Measurement options, result range and precision for “Insight Xpert” urine strip**.**

| **Analysis** | **Range** | | | | | | | **Precision (P) Detection limit (DL)** |
| --- | --- | --- | --- | --- | --- | --- | --- | --- |
| **Glucose^a^** | 0 | 1+ | 2+ | 3+ | 4+ |  |  |  |
| **mg/dl (mmol/l)** | < 40 (2.0) | 50 (2.8) | 100 (5.5) | 300 (17) | 1000 (55) |  |  | DL: 40mg/dl |
| **Protein^b^** | 0 | 1+ | 2+ | 3+ |  |  |  |  |
| **mg/dl** | < 15 | 30 | 100 | 500 |  |  |  | DL: 15mg/dl |
| **pH^c^** | 5 | 6 | 7 | 8 | 9 |  |  |  |
| **Usg^d^** | 1 | 2 | 3 | 4 | 5 | 6 | 7 |  |
| **g/ml** | 1.000 | 1.005 | 1.010 | 1.015 | 1.020 | 1.025 | 1.030 | P: +/- 0.005 |

Usg, Urine specific gravity.

^a^ The **Glucose** test is a specific glucose-oxidase/peroxidase (GOD/POD) reaction based method. The buffer is 3,3`,5,5` tetramethylbenzidine. It is not affected by the presence of ketones or the pH of urine.

^b^ The semi-quantification of **protein** concentration uses tetrabromophenol blue in a reaction based on the principles of “protein error” of pH indicators. The test is particularly sensitive to albumin.

^c^ The **pH** test is based on a double indicator system with methyl red sodium salt and bromthymol blue that shows the full range of pH in urine.

^d^ **Usg** is measured based on the apparent pKa change of certain pre-treated polyelectrolytes in relation to ionic concentration. In the presence of an indicator, colours vary from deep blue to yellow green in urine with increasing ionic concentration. Bromthymol blue and buffer are used in the reaction. A value were elevated one unit if pH of urine were ≥7 in accordance with the strip guideline.

**Table S3.** Background characteristics of included and excluded women and of women recruited through LNGO’s and MCH’s in Hargeisa city.

|  | **Completers** | | | | | | | | | |  |  | **Excluded/dropouts** | | | |
| --- | --- | --- | --- | --- | --- | --- | --- | --- | --- | --- | --- | --- | --- | --- | --- | --- |
|  | **LNGO’s** | |  | **MCH's** | |  |  |  | **All** | |  |  |  |  |  |  |
|  |  |  |  |  |  |  |  |  |  |  |  |  |  |  |  |  |
|  | **Mean** | **SD** |  | **Mean** | **SD** |  | **p-value** |  | **Mean** | **SD** |  |  | **Mean** | **SD** |  | **p-value** |
| Age (yr) | 31.1 | 13.6 |  | 26.6 | 6.8 |  | 0.12 |  | 30.2 | 12.6 |  |  | 26.8 | 10.1 |  | 0.15 |
| Weight (kg) | 59.3 | 14.1 |  | 65.0 | 10.7 |  | 0.07 |  | 60.4 | 13.6 |  |  | 67.9 | 13.8 | * | 0.12 |
| Height (cm) | 161.1 | 6.1 |  | 161.1 | 4.5 |  | 0.99 |  | 161.1 | 5.8 |  |  | 160.3 | 7.0 | * | 0.70 |
| BMI (kg/m2) | 22.7 | 4.9 |  | 25.0 | 3.9 |  | **0.04** |  | 23.2 | 4.8 |  |  | 26.4 | 5.3 | * | 0.06 |
| Household size (n) | 8.9 | 3.0 |  | 7.3 | 2.9 |  | **0.021** |  | 8.6 | 3.1 |  |  | 8.2 | 2.8 |  | 0.73 |
| Children 0-14 in house (n) | 3.1 | 2.3 |  | 4.0 | 2.2 |  | 0.073 |  | 3.3 | 2.3 |  |  | 3.6 | 2.1 |  | 0.43 |
|  |  |  |  |  |  |  |  |  |  |  |  |  |  |  |  |  |
|  | **Median** | **IQR** |  | **Median** | **IQR** |  |  |  | **Median** | **IQR** |  |  | **Median** | **IQR** |  |  |
| Number of childbirths | 0 | 0-7 |  | 4.5 | 2-8 |  | **0.013** |  | 2.5 | 0-8 |  |  | 2 | 1-9 | * | 0.50 |
|  |  |  |  |  |  |  |  |  |  |  |  |  |  |  |  |  |
|  | **%** | **n** |  | **%** | **n** |  |  |  | **%** | **n** |  |  | **%** | **n** |  |  |
| Chronic health problem | 44.7 | 42 |  | 50.0 | 12 |  | 0.81 |  | 45.8 | 54 |  |  | 41.4 | 12 |  | 0.83 |
| 1 or 2 meals /trial | 13.8 | 13 |  | 8.3 | 2 |  | 0.73 |  | 12.7 | 15 |  |  | 22.2 | 2 |  | 0.35 |
| 3 or more meals | 86.2 | 81 |  | 91.7 | 22 |  |  |  | 87.3 | 103 |  |  | 77.8 | 7 |  |  |
| Never attended school | 52.1 | 49 |  | 33.3 | 8 |  | 0.16 |  | 48.3 | 57 |  |  | 48.5 | 16 |  | 1.00 |
| Divorced/widowed | 9.6 | 9 |  | 4.2 | 1 |  |  |  | 8.5 | 10 |  |  | 6.3 | 2 |  |  |
| Single | 51.1 | 48 |  | 0.0 | 0 |  | **<0.005** |  | 40.7 | 48 |  |  | 34.4 | 11 |  | 0.64 |
| Married | 39.4 | 37 |  | 95.8 | 23 |  |  |  | 50.8 | 60 |  |  | 59.4 | 19 |  |  |
| Man head of household | 63.8 | 60 |  | 91.7 | 22 |  | 0.017 |  | 69.5 | 82 |  |  | 87.9 | 29 |  | 0.058 |
| Ahmed Dhagax distr. | 1.1 | 1 |  | 95.8 | 23 |  |  |  | 20.3 | 24 |  |  | 38.2 | 13 |  |  |
| M. Hayb/M Mooge distr. | 36.2 | 34 |  | 4.2 | 1 |  |  |  | 29.7 | 35 |  |  | 20.6 | 7 |  |  |
| Gacan Libaax distr. | 0.0 | 0 |  | 0.0 | 0 |  | † |  | 0.0 | 0.0 |  |  | 0.0 | 0 |  | † |
| 26th June distr. | 17.0 | 16 |  | 0.0 | 0 |  |  |  | 13.6 | 16 |  |  | 14.7 | 5 |  |  |
| Ibrahim Koodbuur distr. | 45.7 | 43 |  | 0.0 | 0 |  |  |  | 36.4 | 43 |  |  | 26.5 | 9 |  |  |
| Hut | 30.9 | 29 |  | 12.5 | 3 |  |  |  | 27.1 | 32 |  |  | 44.5 | 4 |  |  |
| Iron sheet house | 29.8 | 28 |  | 37.5 | 9 |  | 0.20 |  | 31.4 | 37 |  |  | 11.1 | 1 |  | † |
| Cement/brick/Stone-house | 39.4 | 37 |  | 50.0 | 12 |  |  |  | 41.5 | 49 |  |  | 44.5 | 4 |  |  |
| Electricity in house | 50.0 | 47 |  | 37.5 | 9 |  | 0.39 |  | 47.5 | 56 |  |  |  |  |  |  |
| Total **n** |  | 94 |  |  | 24 |  |  |  |  | 118 |  |  |  | 33 | *(9) |  |

LNGO, Local Non-Governmental Organization. MCH, Mother Child Clinic. IQR, Inter quartile range.

Minimum schooling defined as a six months full time course or a successful literacy course

* based on N = 9

† p-values unreliable

**Table S4.** Interrelated hydration index of Uvol, 24h urine units, Usg, Ucol and TFI displaying mean values of seven percentile groups and overall mean for the whole group.

| **Percentiles** | | **Uvol (L)** | | **24h urine units** | | **Usg** | | **Ucol** | | **TFI (L)** | |
| --- | --- | --- | --- | --- | --- | --- | --- | --- | --- | --- | --- |
| **Group** |  | **Mean** | **+/- SD** | **Mean** | **+/- SD** | **Mean** | **+/- SD** | **Mean** | **+/- SD** | **Mean** | **+/- SD** |
| **7** | **91-100** | 2.56 | 342 | 18.8 | 4.58 | 1.006 | 0.0024 | 2.0 | 0.5 | 3.16 | 1068 |
| **6** | **76-90** | 1.89 | 177 | 14.6 | 2.62 | 1.008 | 0.0031 | 2.2 | 0.4 | 2.51 | 873 |
| **5** | **61-75** | 1.44 | 83 | 11.8 | 1.66 | 1.009 | 0.0029 | 2.9 | 0.9 | 2.34 | 747 |
| **4** | **40-60** | 1.16 | 97 | 9.7 | 2.01 | 1.011 | 0.0039 | 3.7 | 1.0 | 1.83 | 724 |
| **3** | **25-39** | 0.94 | 46 | 8.7 | 1.75 | 1.013 | 0.0037 | 4.2 | 1.2 | 1.93 | 558 |
| **2** | **10-24** | 0.74 | 67 | 7.3 | 1.24 | 1.016 | 0.0047 | 5.0 | 1.0 | 1.42 | 612 |
| **1** | **0-9** | 0.45 | 145 | 6.2 | 1.78 | 1.021 | 0.0065 | 5.8 | 1.1 | 1.28 | 528 |
| **Mean** | **0-100** | 1.28 | 605 | 10.8 | 4.26 | 1.012 | 0.0056 | 3.6 | 1.5 | 2.04 | 896 |
| **n** | | 118 | | 118 | | 90 | | 116 | | 118 | |
